# Automated artificial intelligence-based analysis of skeletal muscle volume predicts overall survival after cystectomy for urinary bladder cancer

**DOI:** 10.1186/s41747-021-00248-8

**Published:** 2021-11-19

**Authors:** Thomas Ying, Pablo Borrelli, Lars Edenbrandt, Olof Enqvist, Reza Kaboteh, Elin Trägårdh, Johannes Ulén, Henrik Kjölhede

**Affiliations:** 1 Region Västra Götaland, Department of Urology, Sahlgrenska University Hospital, Göteborg, Sweden; 2 Region Västra Götaland, Department of Clinical Physiology, Sahlgrenska University Hospital, Göteborg, Sweden; 3 Department of Molecular and Clinical Medicine, Institute of Medicine, Sahlgrenska Academy, University of Gothenburg, Göteborg, Sweden; 4 Department of Electrical Engineering, Chalmers University of Technology, Göteborg, Sweden; 5 Eigenvision AB, Malmö, Sweden; 6 Department of Clinical Physiology and Nuclear Medicine, Lund University and Skåne University Hospital, Malmö, Sweden; 7 Wallenberg Centre for Molecular Medicine, Lund University, Lund, Sweden; 8 Department of Urology, Institute of Clinical Sciences, Sahlgrenska Academy, University of Gothenburg, Göteborg, Sweden

**Keywords:** Image analysis (computer-assisted), Body composition, Sarcopenia, Artificial intelligence, Urinary bladder cancer

## Abstract

**Background:**

Radical cystectomy for urinary bladder cancer is a procedure associated with a high risk of complications, and poor overall survival (OS) due to both patient and tumour factors. Sarcopenia is one such patient factor. We have developed a fully automated artificial intelligence (AI)-based image analysis tool for segmenting skeletal muscle of the torso and calculating the muscle volume.

**Methods:**

All patients who have undergone radical cystectomy for urinary bladder cancer 2011–2019 at Sahlgrenska University Hospital, and who had a pre-operative computed tomography of the abdomen within 90 days of surgery were included in the study. All patients CT studies were analysed with the automated AI-based image analysis tool. Clinical data for the patients were retrieved from the Swedish National Register for Urinary Bladder Cancer. Muscle volumes dichotomised by the median for each sex were analysed with Cox regression for OS and logistic regression for 90-day high-grade complications. The study was approved by the Swedish Ethical Review Authority (2020-03985).

**Results:**

Out of 445 patients who underwent surgery, 299 (67%) had CT studies available for analysis. The automated AI-based tool failed to segment the muscle volume in seven (2%) patients. Cox regression analysis showed an independent significant association with OS (HR 1.62; 95% CI 1.07–2.44; *p* = 0.022). Logistic regression did not show any association with high-grade complications.

**Conclusion:**

The fully automated AI-based CT image analysis provides a low-cost and meaningful clinical measure that is an independent biomarker for OS following radical cystectomy.

## Key points

- This fully automated artificial intelligence-based image analysis could segment skeletal muscle volume in almost all the abdominal CT studies.
- The automatically calculated skeletal muscle volume predicted overall survival after radical cystectomy for bladder cancer.
- Further studies are needed to determine optimal volume limits and thresholds for determining sarcopenia.

## Background

Radical cystectomy with or without neoadjuvant chemotherapy is the standard treatment for localised muscle-invasive bladder cancer but is associated with 90-day mortality rate up to 8% and > 50% early complications (within 90 days) [1]. Identifying prognostic biomarkers for these outcomes is therefore important for clinicians and patients in the decision-making.

In recent years, sarcopenia has been found to increase the risk of both cancer-specific mortality and all-cause mortality after radical cystectomy and has also been shown to be an independent predictor of 90-day mortality and complications [2–4]. Sarcopenia is often defined by a low cross-sectional skeletal muscle area in a single slice on computed tomography (CT), most commonly at the level of the third lumbar (L3) vertebra where the cross-section area generally correlates with the total skeletal muscle mass [5]. However, this single-slice area only provides an estimation of the total muscle mass and may not be accurate for all patients. So ideally, as much as possible of the muscle volume should be measured to provide an individual assessment of sarcopenia for each patient. This is a time-consuming process for the radiologist and is subject to intra- and inter-reviewer variability.

Artificial intelligence (AI)-based image analysis models have been developed which can accurately segment muscle and adipose tissue, both in single cross-sections [6] and in full abdominal CT scans [7]. We have previously reported on an AI-based, fully automated image analysis of abdominal CT images, which identifies skeletal muscle and subcutaneous fat and calculates the respective volumes [8]. These volumes have been shown to correlate with the respective area of a single cross-section slice, but with a lower variance and could therefore potentially provide a more reliable measure of sarcopenia. The aim of this study was to assess if this fully automated analysis can be used to predict overall survival (OS) and complications in patients undergoing radical cystectomy.

## Methods

### Patients and study procedure

All patients who had undergone radical cystectomy and urinary diversion for bladder cancer at Sahlgrenska University Hospital in Gothenburg, Sweden from 2011 to 2019, and who had a CT of the abdomen within 90 days prior to surgery, were included in the study. The study was approved by the Swedish Ethical Review Authority (2020-03985). Patient and tumour characteristics, details of complications, and survival status were collected from the Swedish National Registry for Urinary Bladder Cancer [9]. All pre-operative CT studies of the patients included in the study were uploaded to a dedicated platform (recomia.org) for automated image analysis [10]. As part of the uploading process, the image data was stripped of most metadata, except that which was needed for image processing, in order to comply with local laws. Unenhanced image series were used for the image analysis.

The AI-based tool consisted of a single convolutional neural network (CNN), that had previously been trained to segment muscle and fat [8]. In addition, the CNN had also previously been trained to detect and segment the sacrum and coccyx. These skeletal parts were used for determining the levels for muscle segmentation, which is a difference from [8] which used the 11th thoracic vertebra (Th11). The reason for this is twofold. Firstly, the previous determination of level was dependent on all vertebrae being visualised, which was not the case for CT scans depicting only the abdomen. Secondly, the previous determination of level failed in a relatively large proportion of scans, whereas segmentation of the sacrum was more reliable. All skeletal muscle visible on the CT scans was segmented using the automated AI-based tool and then clipped caudally at the caudal tip of the coccyx and cranially at 25 cm cranial to the sacral promontory. The corresponding volume was calculated using the CT slice thickness. Lastly, the accuracy of the segmentations of all the patients was checked by one of the authors (T.Y.), but no manual corrections were made. If the segmentation was grossly incorrect the patient was excluded from the study, while minimal errors were allowed without further correction. There was no exact definition of an incorrect segmentation, all the segmentations were either very good or had almost no part of the muscle volume delineated. The CT studies that were analysed had been performed with a variety of CT scanners with different imaging parameters and at several different radiology departments. No manual annotations had been made to these studies, all were uploaded to the study platform “as-is” and no further annotations were made on the study platform. The AI-based tool was written in the Python language and uses the TensorFlow library for deep learning. Analysing a CT scan takes about 1 min on a high-end desktop computer, depending on the resolution and field of view.

### Statistical analysis

Continuous data were described as median and interquartile range (IQR), while categorical data were described as proportions. For regression analysis, the calculated muscle volumes were dichotomised into below or above the median and also divided into quartiles. Since muscle mass differs for men and women, this was done separately for each sex and then merged for the whole cohort, *i.e*., the lowest quartile for males was merged with the lowest quartile for females, etc. OS ended on death or was censured on November 13, 2020. The main end-point, OS, was analysed by Cox regression analysis with a 2-year restricted follow-up. The variables analysed were age, sex, American Society of Anesthesiologists (ASA) score, smoking, cT stage, previous surgery or radiotherapy in the pelvis, neoadjuvant chemotherapy and year of surgery. For multivariable analysis, the possible predictive variables were added to the model in a step-wise manner. Hazard ratios (HR) were calculated for each of these with 95% confidence intervals (CI). A secondary end-point of any complication of Clavien-Dindo [11] grade 3 or higher within 90 days of surgery was analysed with logistic regression analysis. The possible predictive variables that were analysed were age, sex, ASA score, smoking, type of deviation, previous surgery or radiotherapy in the pelvis, neoadjuvant chemotherapy, clinical local tumour stage (cT) and year of surgery. Odds ratios (OR) were calculated for each of these, with 95% CI. To test for differences in complication rates between the patients who died during follow-up and those who survived until the end of follow-up, *χ*^2^ analysis was done. R version 3.6.2 (R Foundation for Statistical Computing, Vienna, Austria) [12] with the packages tidyverse (1.3.1) [13], survival (3.2-11) [14], and survminer (0.4.9) [15] were used for statistical analysis and visualisation.

## Results

**Fig. 1:**
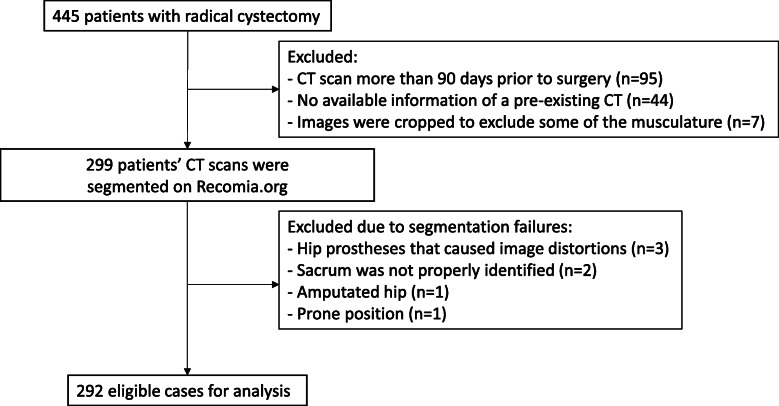
Flow chart of inclusion and exclusions of patients in the study. *CT* computed tomography

Of the entire cohort of 445 patients who had a cystectomy for urinary bladder cancer at Sahlgrenska University Hospital from 2011 to 2019, 299 (67%) patients had a CT study with complete images available within 90 days prior to surgery and were analysed by the AI (Fig. 1). After checking the accuracy of the segmentations, seven (2%) patients were excluded. The AI failed muscle segmentation in three patients due to image distortions from hip prostheses, in two due to failure to identify the sacrum, in 1 due to an amputated hip, and in one where the patient was in the prone position which the AI has not been trained for. The remaining 292 patients were included in the analysis and are detailed in Table 1.

**Table 1:** Clinical characteristics of the patients included in the study

Patient characteristics	Total
Number of patients	292
Age in years, median (IQR)	74 (68–80)
Follow-up time in years, median (IQR)	3.8 (1.4–6.3)
Sex, number (%)	
Male	222 (76)
Female	70 (24)
Smoking, number (%)	57 (20)
ASA score, number (%)	
1	34 (12)
2	174 (60)
3	79 (27)
4	3 (1)
Clinical local tumour stage, number (%)	
CIS	25 (9)
Ta or T1	58 (20)
T2	143 (49)
T3	50 (17)
T4	16 (5)
Clinical node tumour stage, number (%)	
N0	284 (97)
N1	3 (1)
N2 or N3	5 (2)
Extent of LND, number (%)	
None	51 (18)
Only enlarged nodes	19 (7)
Obturator fossa	39 (13)
To the iliac bifurcation	181 (62)
To the aortic bifurcation	2 (1)
Urinary diversion, number (%)	
Ileal conduit	264 (90)
Neobladder	7 (2)
Other	21 (7)
Time between CT scan and cystectomy, days, median (IQR)	48 (33–68)
Neoadjuvant chemotherapy, number (%)	26 (9)
Previous surgery or radiotherapy, number (%)	72 (25)
Highest grade 90-day complication, number (%)	
Grade 0−2	74 (25)
Grade 3	48 (16)
Grade 4	4 (1)
Grade 5	4 (1)
Died during follow-up, number (%)	160 (55)
Died during first 2 years of follow-up, number (%)	95 (33)

*ASA* American Society of Anesthesiologists, *IQR* Interquartile range, *LND* Lymph node dissection

**Fig. 2:**
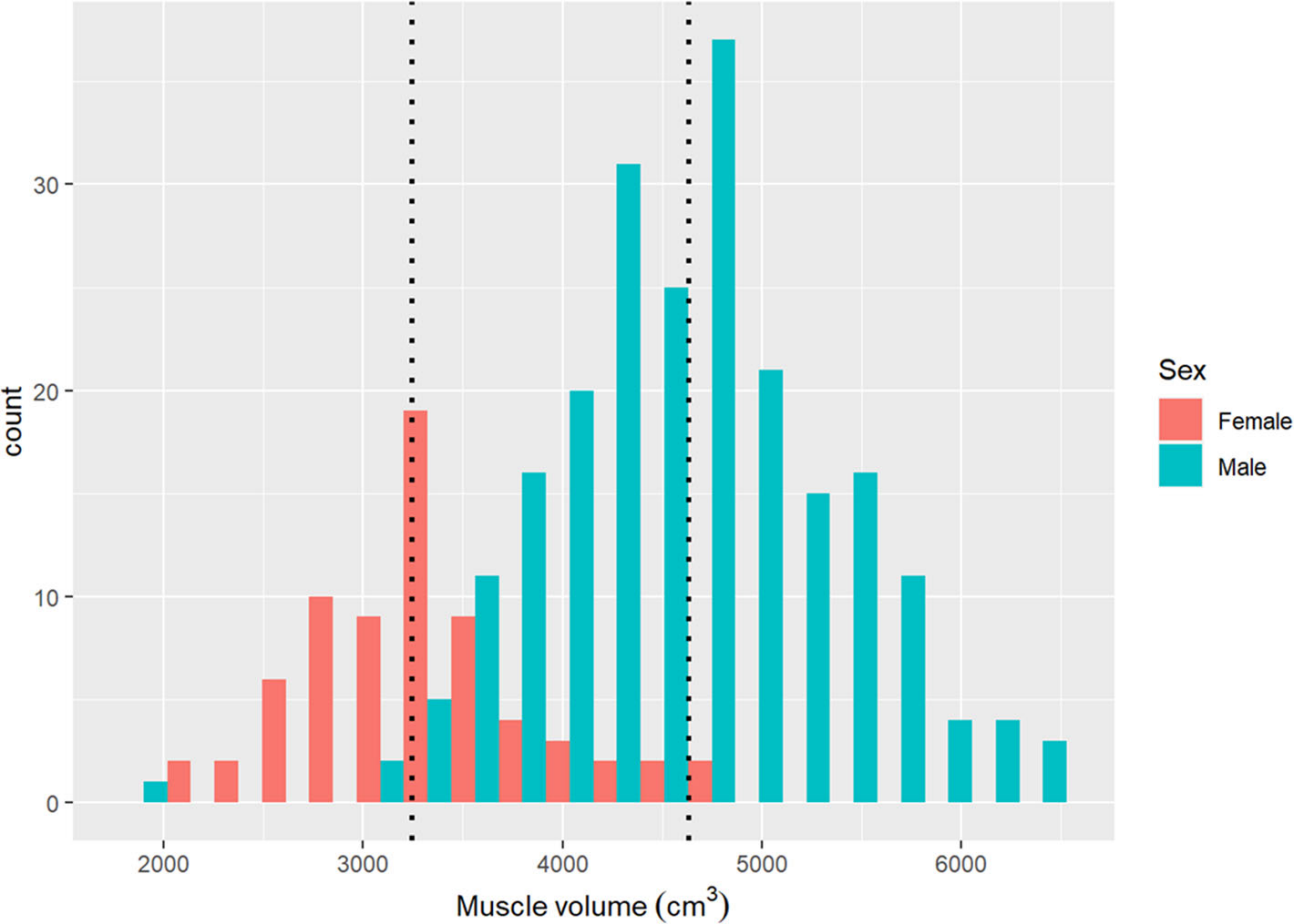
Histogram of calculated muscle volumes, grouped by sex. The median muscle volume was 3,243 cm^3^ for females and 4,629 cm^3^ for males (dotted lines)

The CT scans had been done with 120 kVp in 268 (92%) patients, 100 kVp in 21 (7%), and 135–140 kVp in 3 (1%). Slice thickness was < 3 mm in 46 (16%) patients, 3 mm in 99 (34%), 3–4 mm in 17 (6%), 5 mm in 126 (43%), and 6.5 mm in 4 (1%). The median in-plane resolution of the CT scans was 0.72 (IQR 0.70–0.78) mm. The median calculated muscle volume was 3243 (IQR 2,886–3,600) cm^3^ for females and 4,629 (IQR 4,165–5,039) cm^3^ for males (Fig. 2).

**Fig. 3:**
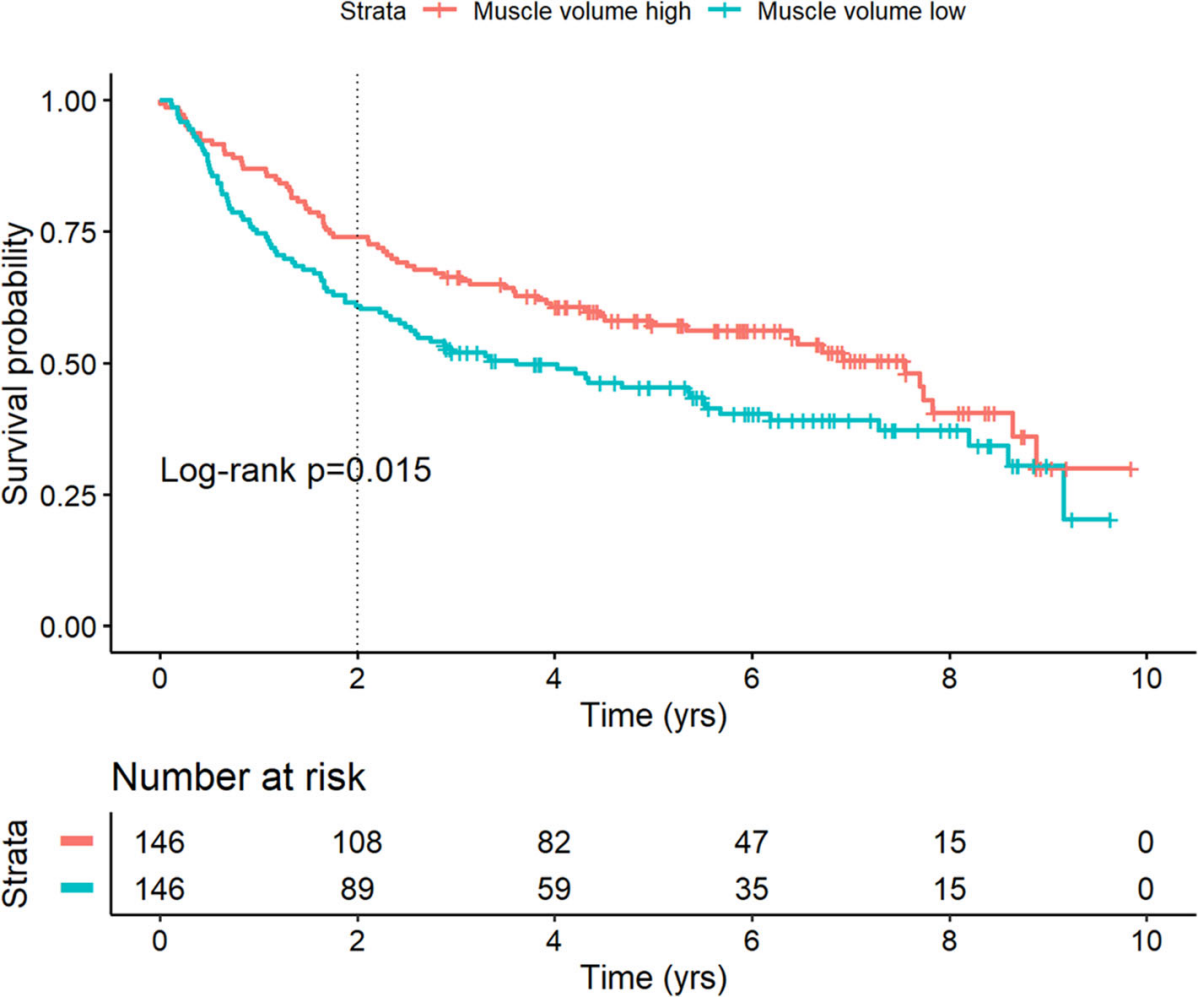
Kaplan-Meier diagram of overall survival grouped by segmented muscle volume higher or lower than the median. Time in years after cystectomy. Log-rank test was done for 2-year restricted overall survival (dotted line), but the diagram shows that the initial difference in survival extends through at least the first 7 years of follow-up

The total mortality rate was 55% (160/291) and the median survival time from surgery among those who died was 3.8 (IQR 1.4–6.3) years. The median follow-up time among those who survived to the end of the study period was 6.0 (IQR 4.6–7.4) years. Figure 3 shows a Kaplan-Meier diagram for OS stratified by muscle volume over or below the median. Of the clinical variables known before surgery, only advanced local tumour stage, high ASA, and low muscle volume were associated with higher overall mortality on univariable Cox regression analysis (Table 2). Analysing these three variables in a multivariable Cox regression showed that all three were independently associated with a higher overall mortality (multivariable model 1). To assess whether there was a correlation between the degree of sarcopenia and mortality, the same variables were analysed but with muscle volumes divided into quartiles. Again, the lowest quartile was independently associated with higher mortality, but not the second-lowest quartile (multivariable model 2). Further, standardising the muscle volume by sex by multiplying the female volumes by the ratio of the medians of both sexes, showed a significant independent linear correlation with OS (HR 0.67 per 1,000 cm^3^; 95% CI 0.51–0.89; *p* = 0.005).

**Table 2:** Cox regression analysis for overall survival with a restricted 2-year follow-up

	Univariable		Multivariable model 1		Multivariable model 2	
	HR (95% CI)	*p*	HR (95% CI)	*p*	HR (95% CI)	*p*
Age, years	1.01 (0.98–1.03)	0.676				
Sex male	1.18 (0.72–1.93)	0.512				
Smoking	1.09 (0.66–1.81)	0.728				
ASA score 3–4	*2.18 (1.45–3.28)*	*< 0.001*	*2.35 (1.56–3.55)*	*< 0.001*	*2.47 (1.63–3.73)*	*< 0.001*
cT stage 2–4	*3.15 (1.91–5.21)*	*< 0.001*	*3.24 (1.96–5.37)*	*< 0.001*	*3.50 (2.11–5.81)*	*< 0.001*
Neoadjuvant chemotherapy	*1.19 (0.62–2.29)*	*0.608*				
Previous pelvic surgery or radiotherapy	*1.08 (0.68–1.70)*	*0.747*				
Muscle volume below median	1.66 (1.10–2.50)	0.016	*1.62 (1.07–2.44)*	*0.022*		
Muscle volume						
Quartile 4	1 (ref)				1 (ref)	
Quartile 3	0.93 (0.49–1.76)	0.821			0.99 (0.52–1.87)	0.964
Quartile 2	1.24 (0.68–2.26)	0.480			1.12 (0.62–2.05)	0.704
Quartile 1	*1.99 (1.14–3.46)*	*0.015*			*2.26 (1.30–3.95)*	*0.004*

ASA score was dichotomised 1–2 *versus* 3–4. cT stage was dichotomised muscle invasive *versus* noninvasive. Muscle volumes were dichotomised higher *versus* lower than median values, and grouped into quartiles, for men and women separately. *ASA* American Society of Anesthesiologists, *CI* Confidence interval, *cT* Clinical local tumour stage, *HR* Hazard ratio

The rate of complications with Clavien-Dindo grade 3 or higher within 90 days of surgery was 20% (56/282). There was no difference in complication rates between those who died during follow-up and those who survived to the end of follow-up (*χ*^2^ *p* = 0. 423). On logistic regression analysis, none of the analysed pre-operative variables were significantly associated with high-grade complications. Similarly, muscle volume below the median was not associated with high-grade complications (OR 1.30, 95% CI 0.73–2.32; *p* = 0.380). Comparing the highest and lowest quartiles of muscle volume also did not show any differences in high-grade complications.

## Discussion

In the present study, we found that the fully automated AI-based image analysis software was able to segment the skeletal muscle volume in over 97% of patients planned to undergo radical cystectomy for urinary bladder cancer. The calculated muscle volume was independently associated with restricted 2-year OS. The calculated muscle volume was not, however, associated with high-grade complications in the first 90 days following surgery.

The independent association between low muscle volume and OS found in the present study is in line with the study by Psutka et al. [2], which found a HR of 1.93 in overall mortality for patients with sarcopenia. Similarly, Mayr et al. [4] found a HR of 1.43 for the same outcome. In contrast, Smith et al. [16] did not find a statistically significant association with 2-year OS, although that may have been due a low statistical power with only 200 patients included compared to 500 in Mayr et al. [4], 205 in Psutka et al [2] and 291 in the present study.

Taken together, the studies do seem to suggest that there is a clear association between sarcopenia and OS following radical cystectomy for urinary bladder cancer. The main difference between these studies and the present one is the analysis of muscle volume compared to skeletal muscle area in a single axial cross-section at the level of the L3 vertebra. We have previously shown that these different measurements correlate, but that muscle volume has a lower variance and may therefore be a better and more reliable measure of sarcopenia [8]. The present study builds on that by showing that only very few CT studies could not be correctly analysed and that the association with OS holds true. Specifically, we analysed 2-year OS, but Kaplan-Meier analysis showed that the difference in OS persisted up to 7 years after surgery, after which there were very few patients at risk.

In our study, we did not find an association between sarcopenia and high-grade complications. This is in contrast to Mayr et al. [3] which did find a significant association between L3 cross-section muscle area and high-grade complications. That study had a larger proportion of grade 4 and 5 complications than our study (12% and 3%, respectively), which may explain that they were able to find this association. Differing definitions of sarcopenia could also have been a possible explanation; however, there were no significant differences between the highest and lowest quartiles in our study suggesting that this was not the case. A further possible explanation may be that we have had a protocol for enhanced recovery after surgery in place for radical cystectomy for most of the study period. This may have decreased the number of high-grade complications leading to a low power to detect an association [17]. Nevertheless, it is possible that this automated analysis of sarcopenia could be used to evaluate if patients require further interventions to prevent complications after surgery. This becomes especially important when considering recently updated guidelines recommending radical cystectomy for more elderly patients with non-muscle invasive bladder cancer, that has a relatively better prognosis than those included in the present study [18].

In our study, the muscle volume was calculated from the tip of the coccyx and 25 cm cranial to the sacral promontory. This arbitrary limit was chosen a priori because of limitations in the CT studies that had been performed for these specific patients. However, technically the calculation can easily cover the entire torso if such CT studies are available. For muscle area in a single L3 axial cross-section, the lumbar skeletal muscle index (SMI, the area of the muscles divided by the patient body height squared) is the most often used measure for sarcopenia, with different cutoffs for men and women and in different population [19, 20]. For muscle volume there exist no definitions for sarcopenia that could be used, which is a major limitation for this study, and any such definitions are necessarily dependent on which sections of the torso that are analysed. The choice of measuring muscle volume was made based on our previous work showing that the muscle volume has a lower variability than L3 cross-section area and should therefore be more useful in sarcopenia assessment. Further research is needed both to elucidate the optimal sections for analysis and what thresholds best describe sarcopenia. However, an advantage of the automated analysis tool that we have developed is that the image analysis does not require any manual input and such research can thus be performed with very little additional resources [21]. Furthermore, sarcopenia can also be defined in terms of skeletal muscle quality [22], and it may be possible to identify image patterns or other radiomic data within the muscle volume that can improve the detection of sarcopenia.

The main limitation of the present study is the lack of a validated muscle volume threshold for determining sarcopenia, which limits the immediate applicability of the tool. Further, the study is retrospective, with all the inherent limitations that entail including the lack of some relevant clinical data and a clinical evaluation of sarcopenia with a validated instrument. The long study period also means that technical improvements in CT scan acquisition could have affected the results. Also, this study, like the other retrospective studies previously described, was not designed to show whether the sarcopenia is treatable. Especially in the context of bladder cancer patients, where the time available before surgery is limited, any such interventions need further study. However, the finding of sarcopenia together with other clinical data could identify patients with such a poor prognosis that other, palliative treatments may be more appropriate than radical cystectomy.

In conclusion, the fully automated AI-based image analysis of CT studies can reliably calculate skeletal muscle volume in patients with urinary bladder cancer. This provides a low-cost and meaningful clinical measure that is an independent biomarker for overall survival following radical cystectomy.

## Data Availability

The datasets used and/or analysed during the current study are available from the corresponding author on reasonable request.

## Abbreviations

AI: Artificial intelligence
ASA: American Society of Anesthesiologists
CI: Confidence interval
CNN: Convolutional neural network
CT: Computed tomography
HR: Hazard ratio
IQR: interquartile range
OR: Odds ratio
OS: Overall survival
